# Dynamic evolution of policy mix in response to COVID-19: Practice from China

**DOI:** 10.1371/journal.pone.0291633

**Published:** 2023-09-28

**Authors:** Lei Du, Wei Lu

**Affiliations:** 1 School of Public Administration, Southwestern University of Finance and Economics, Chengdu, Sichuan Province, China; 2 School of Business, Southwest Minzu University, Chengdu, Sichuan Province, China; Zhejiang Gongshang University, CHINA

## Abstract

COVID-19 is a global pandemic. In response to this unprecedented crisis, Chinese government formulated a series of policies. This research is dedicated to exploring the dynamic evolution of China’s policy mix in response to COVID-19 in different crisis response stages from a network perspective. First, a three-dimensional analysis framework of “policy subject-policy target-policy instrument” was developed. Then, based on the data sets collected by textual analysis, the dynamic evolution of policy subject, policy target, policy instrument in China’s policy mix in response to COVID-19 was discussed by using the method of SNA. This study concluded that the core policy subject, policy instrument, and policy target of China’s response to COVID-19 changed with time. National Health Commission (NHC), Ministry of Finance (MOF), Ministry of Transport (MOT) and Ministry of Human Resources and Social Security (MHRSS) have important influences in the network of policy subjects. Other subjects are more at the edge of the network, and there are few joint issuances among policy subjects. The study also found that the core policy target was adjusted over time, with phased dynamic characteristics. At the initial stage of China’s response to COVID-19, “reduce infection and mortality” and “steadily carry out economic and social work” were the core policy targets. With the COVID-19 under control, “enterprise development and work resumption” becomes a new core policy target. In addition, this study also revealed the dynamic evolution and unbalanced use of China’s policy instruments in response to COVID-19 in different stages. The combination of policy instruments is mainly composed of “mandatory administration instruments” and “economic incentive instruments”, and supplemented by “health promotion instruments” and “voluntary plan instruments”. These findings may enrich the literature on COVID-19 policy to help researchers understand the dynamics of policy from a network perspective. Moreover, these findings may provide several valuable implications for policymakers and other countries to formulate more effective policies for epidemic response.

## 1.0 Introduction

The novel coronavirus (SARS-CoV-2) is a highly infectious viral pneumonia, named Corona Virus Disease 2019 (COVID-19) [1]. In December 2019, COVID-19 suddenly broke out in Wuhan, China, and quickly triggered a historic-scale epidemic in more than 200 countries and territories around the world [2]. This serious public health emergency (PHE) threatens human life and public health security. On January 30, 2020, World Health Organization (WHO) declared COVID-19 as a “public health emergency of international concern (PHEIC)” and escalated it to a global pandemic on March 11 [3]. This unprecedented global public health crisis challenges the national governance system. To contain the rapid spread of COVID-19, Chinese government instituted and implemented a series of aggressive intervention policies, involving health, medical, economic, and welfare [4, 5]. Two months later, China flattened the coronavirus curve and achieved preliminary success in the war against COVID-19 [6]. In sharp contrast, countries outside of China, especially in Europe and the United States, have experienced increasingly serious COVID-19 epidemic since mid-March, and the number of confirmed infections and deaths has continued to increase. As of June 28, 2020, there were more than 10 million reported COVID-19 cases in the world, while less than 1% of confirmed cases in China [7]. Policies implemented by the Chinese government greatly alleviated the epidemic and achieved the most reliable and rapid effect. WHO praised China’s efforts in response to COVID-19. In dealing with such PHEs, criticism and praise can easily point to the national governments and their policies. China’s success in response to COVID-19 represents the success of the government governance and policies adopted.

Policy is an important means of national governance, which is defined as the tool or technology taken by the government to achieve the expected targets [8]. Policies include multiple elements such as the participants, instruments, targets [9]. Research on the policy should expand the scope of analysis from a single policy element to a wider policy mix. Policy mix is a complex arrangement of multiple policy goals and means, which helps to better capture the complex policy elements and their dynamic changes over time in the realistic response to PHEs [10]. Thus, it is a good attempt to explore the China’s response to COVID-19 from the perspective of policy mix.

In recent years, policy mix has received extensive attention from scholars [11]. Using policy texts to qualitatively study the policy mix is the mainstream research way [12, 13]. However, qualitative research depends on the researcher’s knowledge and ability, which means that the reliability and universality of the results may be questioned. This paper bridged this gap by employing textual analysis and social network analysis (SNA) methods to conduct a comprehensive qualitative-quantitative study on China’s policy mix in response to COVID-19. Meanwhile, some recent studies have gone beyond a focus on single policy. Yet they have mostly focused on theoretical discussions or brief overviews, and lack comprehensive research on policy mix in mitigation the COVID-19 pandemic. Thus, this paper advocated an extended concept of the policy mix and developed a three-dimensional analysis framework of “policy subject-policy target-policy instrument” to better explain the structure and element relationship of China’s policy mix against COVID-19 at the micro-level. In addition, time is a crucial dimension in policy mix concept [14] 2020a. While several studies on policy mix of the pandemic have been published recently, such as Ning, et al. (2020), Yan, et al. (2020) [15, 16], most of them are mainly based on a static perspective without considering the dynamic of policy mix in different response stages. Pandemics like COVID-19 need a long time to respond, the policy mix aimed at mitigating the crisis may change over time to adapt to the changing policy scenario. Therefore, this study calls for the integration of temporal dynamics into the research of PHEs policy mix to produce more meaningful insights.

The above criticism and gap provide room for this study. Therefore, this paper aims to explore the core node and dynamic of policy mix in response to COVID-19 in China by SNA. To address this issue, this paper will answer the following questions:

- What are the core policy subjects, policy instruments and policy targets in China’s response to COVID-19 policy mix?
- How do the policy subjects, policy targets, as well as policy instruments evolve over time in different stages of China’s response to COVID-19?
- What experiences and lessons can be learned from China’s response to COVID-19?

## 2.0 Literature review

The outbreak of COVID-19 has become a “focus event”, triggering a discussion on the PHEs emergency policies in academic community. In this paper, a systematic review of its relevant theoretical foundations and research literature was presented.

### 2.1 Theoretical foundation

Policy is the tool or technology taken by the government to achieve the expected targets [8]. That is, policies need to be formulated through the corresponding subjects, as well as implemented with the help of certain instruments to achieve expected policy goals. Thus, policy subjects, policy targets, and policy instruments construct the basic policy research paradigm. Policy subject is the key to the realization of policy targets; Policy target is employed to provide the direction for the policy subjects to select appropriate policy instruments; Policy instrument serve as a major means to achieve policy target [17].

In addition, as a structural theory in policy science, policy instrument has gained popular attention for its strong explanatory power and wide applicability. A variety of policy instrument models have been developed in the academic community. Dahl & Lindblom (1954) pioneered a systematic study of policy instruments, categorizing them into regulatory and non-regulatory instruments from perspectives of mandatory degree [18]. Based on the same classification criteria, Howlett (1991) divided policy instruments into voluntary, mixed and mandatory instruments [19]. This categorization attempts to understand how governments influence the recipients of policies. Differently, Rothwell & Zegveld (1981) weakened the mandatory characteristics of policy instruments and divided policy instruments into three categories of supply, demand, and environmental from perspectives of instrument influence [20]. These studies provide theoretical guidance for the analysis of China’s COVID-19 policy instruments in this paper.

### 2.2 Policy mix and PHEs emergency response

The outbreak of COVID-19 caused an unprecedented public health crisis worldwide. Public policy has responded to this societal pressure. Governments around the world have formulated various policies to address this intractable epidemic challenge, but their effectiveness and results are not the same, which has been intensively studied by scholars. As the country that first broke out and successfully responded to COVID-19, China has achieved exceptional performance. Ning, et al. (2020) summarized China’s policies toward COVID-19, such as government response and accountability, citizens participation in epidemic emergency response, etc., which provide guidance for other settings [15]. On this basis, Lai et al. (2020) developed a modeling framework to simulate different interventions in China [21]. The research results indicated that the effectiveness of different interventions varied, but the combination of non-drug interventions achieved the strongest and most rapid effects. Compared with many other countries, Singapore’s COVID-19 mortality rate is very low, thanks to its strict response policies such as travel bans, contact tracing, and social distancing [22]. Coccia (2023) did not agree with this view [23]. He argued that high-level strict containment policies may not be effective control in containing the spread and negative impact of pandemics like COVID-19. In the battle against COVID-19, the South Korean government also showed a good response. Through reviewing South Korea’s public health policies for controlling COVID-19 outbreaks, You (2020) found that emergency use authorization is an important policy for South Korea to successfully control the epidemic [24]. As early as 2013, Courtney et al. proposed that the new drug applications and emergency use authorizations formulated before emergencies can speed up the development and application of new medical programs during the response period [25]. However, compared with successful cases, the response of the United States to COVID-19 is not satisfactory. Carter & May (2020) sought to explore what impeded the efforts of the United States to respond to the COVID-19 pandemic from regime perspective [26]. The study concluded that lack of political commitment, ambiguous goals, and inertia of Party and economic interests hindered the US response to the virus.

Moreover, beyond the study of a single case, scholars have studied the policy effectiveness of different countries around the world to respond to COVID-19 based on multiple cases. For example, Weng, et al. (2020) compared the anti-epidemic experiences of Shanghai, China and Los Angeles, USA. The study found that the implementation of the “four-early policy” in Shanghai prevented the spread of the virus locally [27]. Similarly, Khan et al. (2021) evaluated the effectiveness of pharmacologic and non-pharmacologic policy measures adopted by seven South Asian countries (Afghanistan, Bangladesh, Bhutan, Nepal, Pakistan, Sri Lanka) to reduce the COVID-19 pandemic [28]. The results concluded that economic support, stringency, and health and containment measures played an important role in reducing the COVID-19 pandemic. Different from the above studies, some literature found that the effectiveness of COVID-19 interventions needs to be interpreted specifically within a particular political, economic and cultural context. For example, Yan, et al. (2020) analyzed the different COVID-19 response policy choices (nudge, mandate, decree, and boost) in Sweden, China, France and Japan, and concluded that the various policies regarding the same threat depend on the unique institutional arrangements of each country [16]. Consistent with this argument, An & Tang (2020) analyzed the COVID-19 policy instruments of five developed economies in East Asia and suggested that policy instruments adapted to basic cultural orientation are more likely to promote public cooperation and voluntary compliance [29]. One important insight of these literatures is that a feasible policy mix is inherently conditioned by various countries’ institutional context, cultural orientation, as well as political styles. Therefore, there is no best policy mix for successful PHEs emergency response, but it should be compatible with the political system and culture of the respective country.

Based on above literature review, it was found that previous studies have focused more on the static assessment of COVID-19 policy effectiveness, while lacked attention to the dynamics of the policy mix. However, research from a static perspective tends to ignore the small changes in the internal structure and relationships of the policy mix, which is not conducive to the analysis of complex policy issues. In the field of emergency management and risk governance, the research on network dynamics based on time slice has become a new way for scholars. Meanwhile, in the choice of research methods, limited by the characteristics of policy texts, some scholars adopt qualitative interpretation methods to reveal the effectiveness of COVID-19 policies. However, as proposed by Howlett & Del Rio (2015), the lack of quantitative research leads to a limited understanding of policy mix [30]. To address this limitation, some scholars have conducted quantitative studies with the help of computer simulation. But, due to the relatively low frequency of public health events occurrence, the accuracy of results based on computer simulation is difficult to verify. A comprehensive study combining qualitative and quantitative methods is urgently needed.

## 3. Methodology

### 3.1 Research design

**Fig 1:**
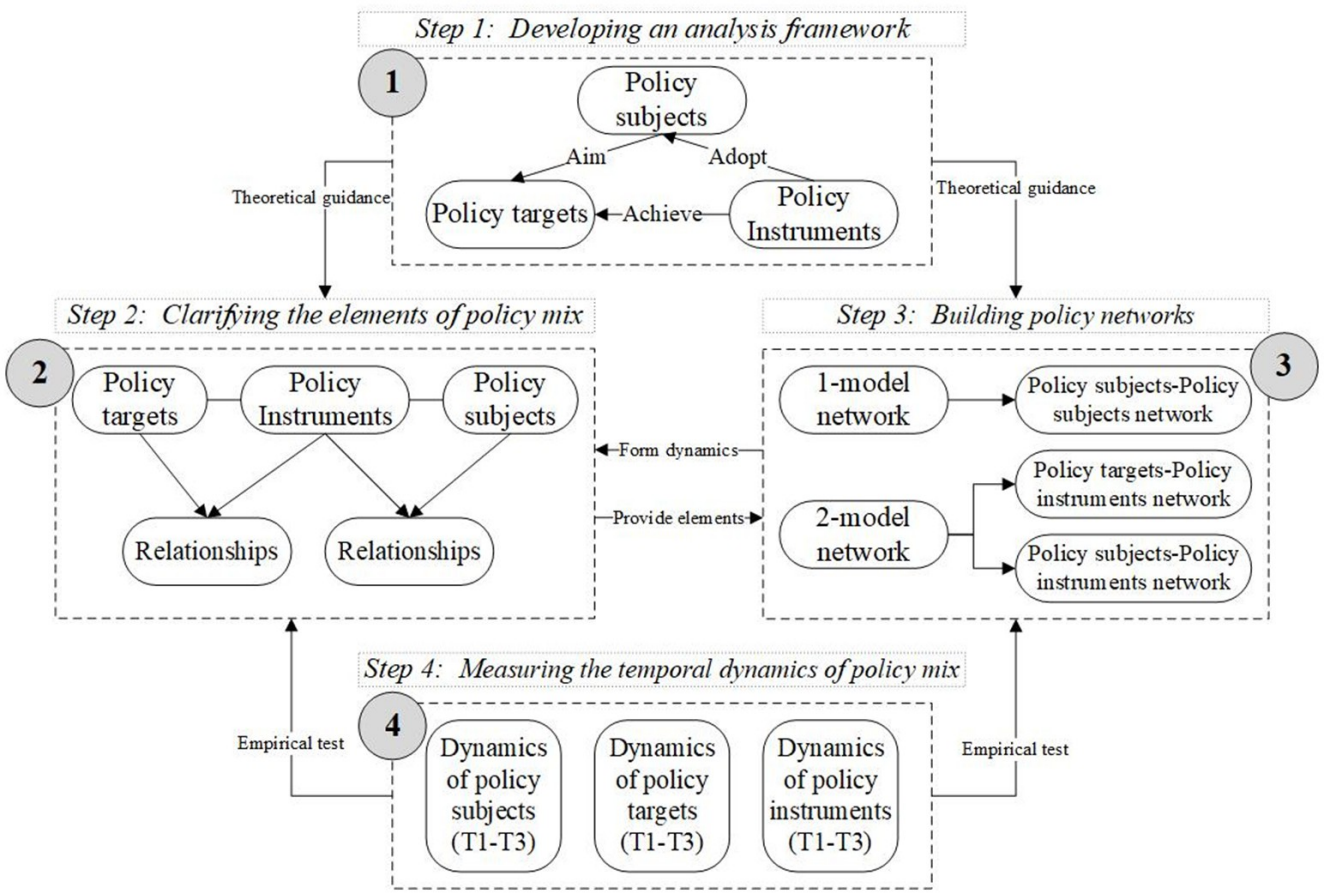
Research strategy.

Three steps were designed to address the issues raised in this paper, as shown in Fig 1. The first step is to develop an analysis framework. Based on previous literature, a three-dimensional analysis framework of “policy subject-policy target-policy instrument” for China’s policy mix in response to COVID-19 was constructed. The second step is to clarify the elements of policy mix. Textual analysis was adopted to determine the policy subjects, policy targets, policy instruments, as well as the relationships among them implied in China’s response to COVID-19 policy texts. The third step is to build policy networks. According to the actual interactions of policy subjects, policy targets and policy instruments, 1-model and 2-model policy networks were built. The fourth step is to measure the temporal dynamics of policy mix composed of policy subjects, policy targets and policy instruments. Core policy subjects, policy targets and policy instruments at different stages were identified by using SNA, and then the dynamic characteristics and evolution rules of China’s policy mix in response to COVID-19 were obtained.

### 3.2 Analysis framework of “Policy subject-policy target-policy instrument”

**Fig 2:**
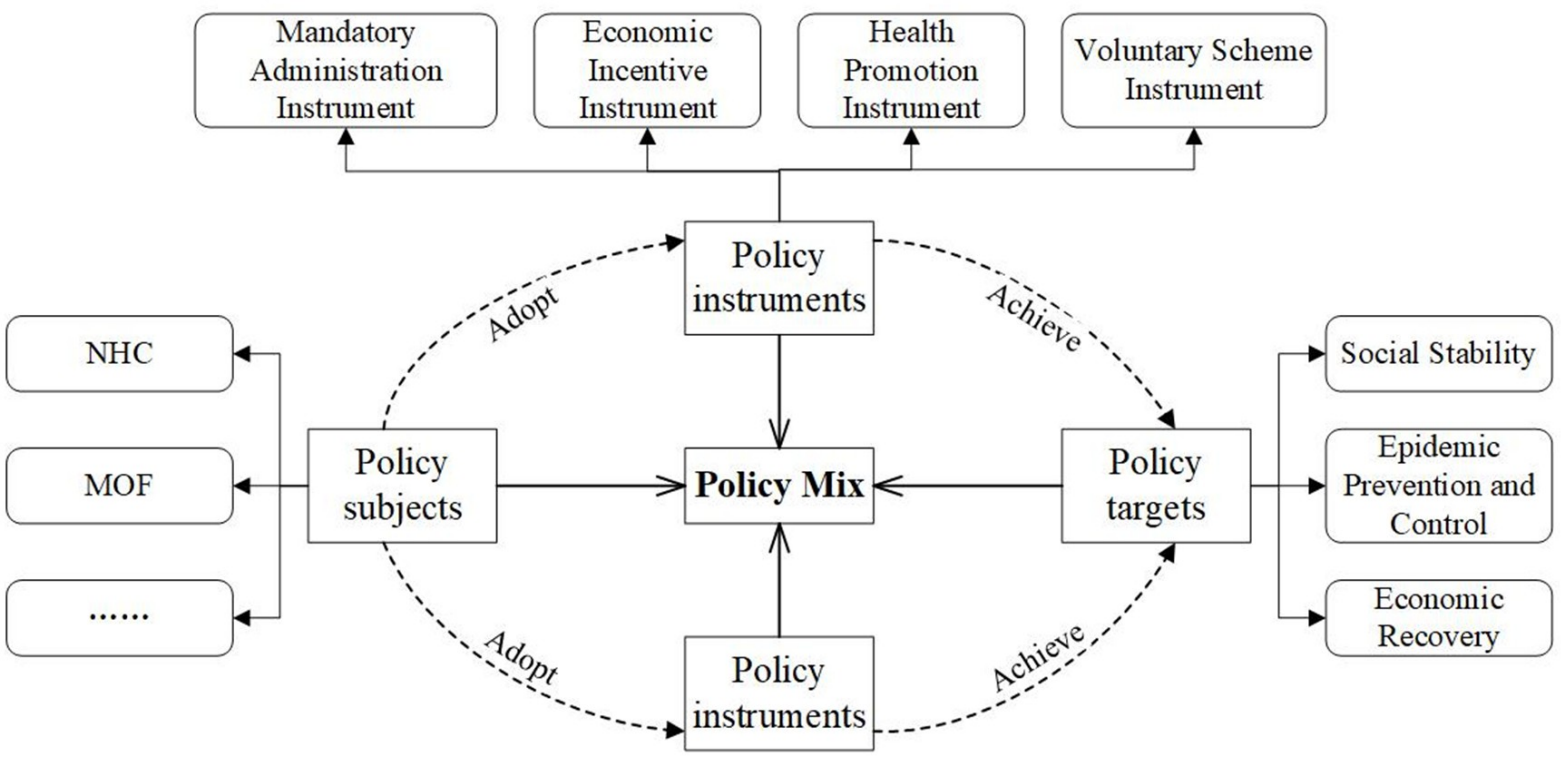
Three-dimensional policy analysis framework.

A reasonable policy analysis framework is the premise to ensure the strength of research interpretation. According to Peter Hall’s policy paradigm theory, policy analysis should comprehensively consider a variety of elements, such as policy subjects, policy targets and policy instruments, these constitute a policy system [31]. Different policy subjects and their matching forms will produce different policy effects; different policy instruments will lead to different policy results; and policy targets may be inconsistent at different stages of the policy implementation process, which requires the combination of policy subjects, policy targets and policy instruments for analysis. These three dimensions are an interconnected whole, forming a new way to interpret the policy mix of China’s response to COVID-19. Accordingly, a three-dimensional analysis framework of “policy subjects-policy targets-policy instruments” was developed in this study, as shown in Fig 2.

The specific content of the three-dimensional analysis framework is as follows. Among them, policy subject is the institution, organization or group that promulgates relevant policies following legal procedures to solve a certain public issue, namely the policy issuing subject. Policy target is the expected effects and purposes of policy formulation and implementation [17]. The direct harm of COVID-19 is the threat to human life and health. Traffic control, lockdown, and production shutdowns during the response to COVID-19 led to economic stagnation. And human health threat and economic crisis posed by COVID-19 also caused social stability issues. Thus, epidemic prevention and control, economic recovery and social stability constitute the policy targets of China’s response to COVID-19. Policy instruments are diversified governance technologies or means implemented by policy subjects to achieve specific targets or solve certain problems [32]. The primary issue of policy instrument research is the division of policy instruments. Many policy instrument classification systems have been developed in academic community. From the perspective of mandatory degree, Howlett identified three types of policy instruments of “mandatory instrument, mixed instrument and voluntary instrument” [19]. Based on the tracking of COVID-19 response policies in major countries around the world, Hale et al. (2020) summarized various government policies into four groups of “containment and closure, economic response, health systems and miscellaneous” [33]. Referring to the classification of policy instruments by Howlett (1991) and Hale et al. (2020), this study divided the PHEs policy instrument into four categories of mandatory administration instrument, economic incentive instrument, voluntary scheme instrument and health promotion instrument.

### 3.3 Data collection and processing

Policy documents are objective, accessible and traceable textual records of government behavior, which provide a reliable data channel for researchers to conduct quantitative research on policy subjects, targets and instruments [12]. As a data source, policy document has its unique advantages. First, policy document can truly reflect the value orientation and policy position of policy subjects that may not be easily obtainable through other methods, such as metrological statistics. Second, policy document retains contextual information, which has great potential for extracting the policy intentions of decisionmakers. Third, policy document is easily accessible. Professional policy data platforms have been established in China, such as the “Peking University Law Database”. In addition, as China’s government information disclosure work continues to deepen, large amounts of policy documents have been provided to the public and researchers for free. Therefore, this paper attempts to use the data generated from policy documents to present the dynamic of China’s response to COVID-19 policy mix.

#### 3.3.1 Data collection

In this research, data was collected by browsing the professional policy database of “Peking University Law”. “Peking University Law” is a comprehensive and widely used policy database that compiles various laws, regulations, and rules promulgated by the central and local governments. The key term “epidemic prevention and control” was used to retrieve the related policies. Finally, 243 valid policy documents (show in Table 1) were obtained by the following principles. (1) The policies issued from January 20, 2020 to March 26, 2020 were collected. On January 21, 2020, the National Health Commission (NHC) of the People’s Republic of China (PRC) promulgated the “Announcement Regarding the Inclusion of Pneumonia Infected by the New Coronavirus into statutory Infectious Diseases Management”, marking the start of the COVID-19 emergency response campaign in China. On March 26, there were no new local cases in many provinces, which meant that China has achieved initial success in the fight against COVID-19. (2) The policy documents are limited to policies of the central government, while the policies of local governments were not included. In the face of major PHEs such as COVID-19, the Communist Party of China (CPC) Central Committee and the central government quickly deployed. And local governments are subordinate to the central government under China’s centralized political system. Thus, the policies issued by the central government are more representative and authoritative, and can better reflect the basic policy concept of epidemic emergency response in China. (3) The policy content belongs to the thematic classification of “emergencies”, which is highly related to COVID-19’s emergency response. (4) The types of policies mainly include laws, administrative regulations, departmental rules, etc.

**Table 1:** Policy documents for COVID-19 emergency response in China.

No.	Issuing Time	Issuing Agencies	Name of Policy Documents
1	January 31, 2020	MOF	Notice of the Ministry of Finance on Further Doing a Good Job in Guaranteeing Funds for the Emergency management of the Novel Coronavirus Pneumonia Outbreak
2	February 3, 2020	NHC	Notice of the General Office of the National Health Commission on Strengthening Informationization to Support the Emergency management of the Novel Coronavirus Pneumonia Epidemic
3	January 29, 2020	MOT	Urgent Notice of the Ministry of Transport on Coordinating the Epidemic Emergency management and Transportation Guarantee Work
4	February 3, 2020	MOC	Notice of the General Office of the Ministry of Commerce on Further Facilitating Technology Import and Export During the Period of Epidemic Emergency management
5	February 14, 2020	MIIT	Notice of the General Office of the Ministry of Industry and Information Technology on Further Doing a Good Job in the Emergency management of the Novel Coronavirus Pneumonia Epidemic
…	…	…	…
239	February 14, 2020	SAMR	Guiding Opinions of the General Administration of Market Regulation on Investigating and Punishing Illegal Acts of Raising Prices during the Emergency management of the Novel Coronavirus Infection
240	February 14, 2020	NHC	Notice of the General Office of the National Health Commission on Issuing Guidelines for the Emergency management of the New Coronary Pneumonia Epidemic of Occupational Health Technical Service Institutions
241	February 16, 2020	NHC MOCA	Notice of the Ministry of Civil Affairs and the National Health Commission on in-depth Study and Implementation of the Spirit of General Secretary Xi Jinping’s Important Instructions to Further Improve the Emergency management of the Epidemic in Urban and Rural Communities
242	February 21, 2020	JPCMSC	Notice of the State Council on the Joint Emergency management Mechanism for Novel Coronavirus Emergency management Measures for Enterprises and Institutions Resuming Work and Production
243	March 11, 2020	MOC	Notice of the Ministry of Commerce on Coordinating the Emergency management of the New Coronary Pneumonia Epidemic and Business Poverty Alleviation

#### 3.3.2 Data processing

Textual analysis is a quantitative analysis method based on qualitative research. It transforms unsystematic and qualitative symbolic content such as text, image, sound, etc. into systematic, quantitative data that can reflect the essence of policy content through coding and statistics. Content analysis has gained validation as a powerful research tool in many policy analysis studies. Therefore, textual analysis was conducted on 243 national-level of COVID-19 emergency response policy documents to extract and refine policy subjects, policy instruments, policy targets, and the relationships between them, including three steps.

**Fig 3:**
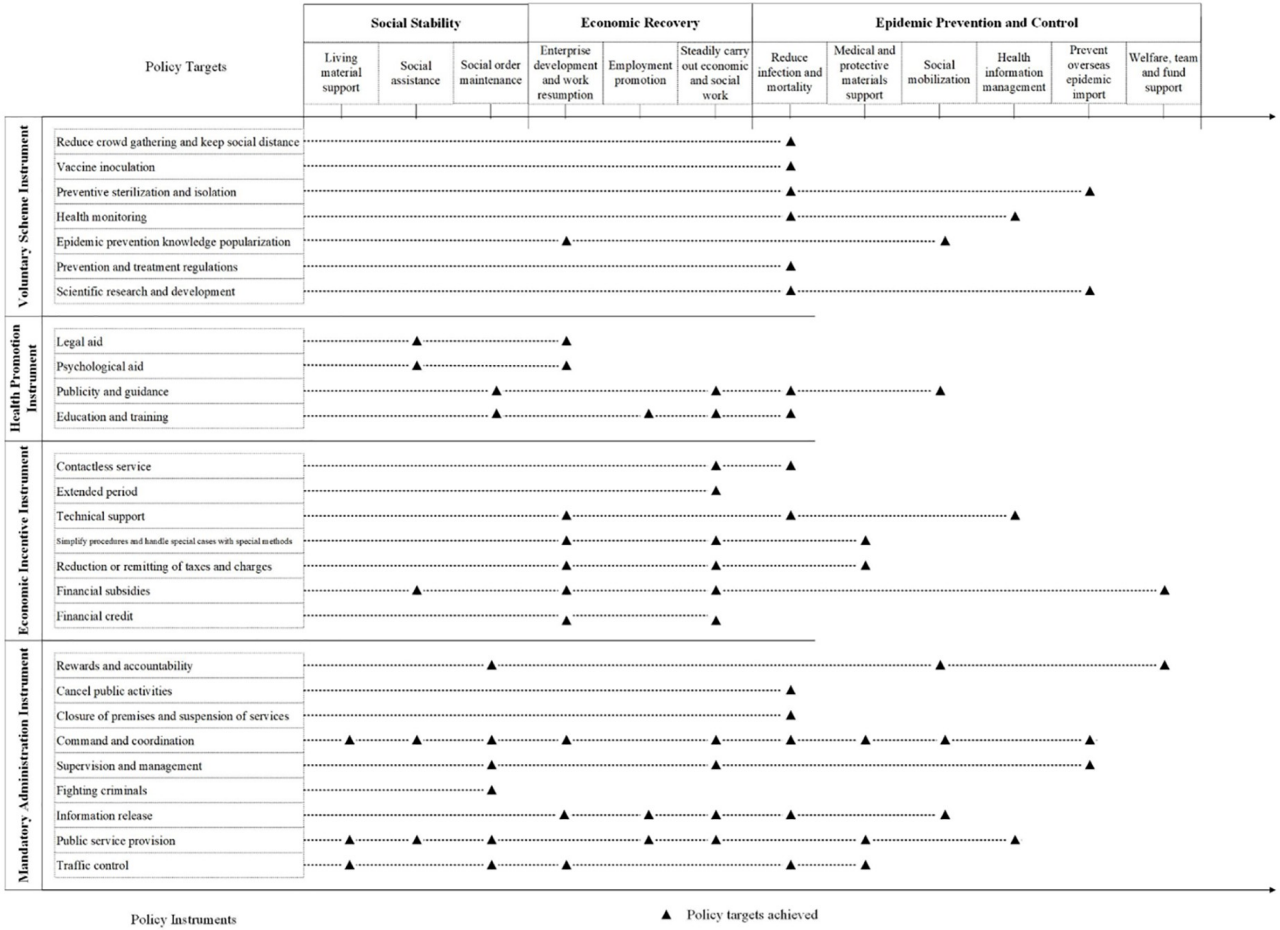
Policy targets and policy instruments.

1. The first step is to clarify the policy subjects, policy targets and policy instruments involved in China’s response to COVID-19. Policy documents in China are written in a highly standardized style. The issuing subject is usually marked before the policy content. With such fixed patterns, 86 policy subjects were easily identified through the manual inspection, as shown in S1 Appendix. One policy target/policy instrument may contain several sub-policy targets/sub-policy instruments. To improve the accuracy of policy target/instrument identification, this paper conducted semantic analysis on the phrases, sentences or paragraphs of the policy texts, and then unified the expression of them (seen in Table 2). As a result, 12 sub-policy targets and 27 sub-policy instruments were refined, as presented in Fig 3.
2. The next step is to extract the relationships among policy subjects, policy targets and policy instruments. Specifically, the interactions were noted by manually mining and reviewing sentences in policy documents regarding China’s response to COVID-19. For example, the interaction between the policy target of “reduce infection and mortality” and the policy instrument of “reduce crowd gathering and keep social distance” was extracted from the sentence of “minimize the gathering and flow of people, and effectively protect the lives, health and safety of the people” in policy document. Consequently, a total of 359 links between policy subjects, 121 links between policy targets and policy instruments, and 295 links between policy instruments and policy subjects were identified in this study.
3. The last step is to construct the multi-valued data matrix. The multi-value matrix is expressed by the existence and strength of the nodes and links, not only indicating whether the relationship exists, but also the interaction strength of the relationship [34]. If there is a joint issuing document between policy subjects; if a policy instrument is used to achieve a policy target; if the policy subject adopts a policy instrument to achieve a policy target, it is coded as “1”, otherwise, coded as “0”. And if there is more than one joint issuing document between the same policy subjects; if the same policy subject adopts the same policy instrument multiple times to achieve the same policy target, the previous code value is added 1. As a result, multi-valued 2-mode data matrixes of “policy subject-policy subject”, “policy instrument-policy target” and “policy subject-policy instrument” were constructed. These data matrixes would be imported into the UCINET software to measure the dynamic of China’s response to COVID-19 policy mix.

**Table 2:** Part of the policy targets/instruments extraction process.

Policy Instrument	Sub-policy Instrument	Text Source	Extract Contents
Mandatory Administration Instrument	Traffic control	Urgent Notice of the Ministry of Transport on Coordinating the Epidemic Emergency management and Transportation Guarantee Work	“According to the unified deployment of the Ministry of Transport, under the leadership of the local Party committee and government, and in combination with the local epidemic emergency management situation, local transportation departments have fully or partially implemented emergency management measures such as suspending the operation of inter-provincial lines in and out of Wuhan and all inter-provincial chartered vehicles.”
Economic Incentive Instrument	Technical support	Notice of the General Office of the National Health Commission on Strengthening Informationization to Support the Emergency management of the Novel Coronavirus Pneumonia Epidemic	“Use big data technology to track, screen and predict the epidemic development in real-time, to provide data support for scientific prevention and precise policy implementation.”
Health Promotion Instrument	Reduce crowd gathering and keep social distance	Notice of the General Office of the National Health Commission on Issuing Guidelines for the Emergency management of the New Coronary Pneumonia Epidemic of Occupational Health Technical Service Institutions	“Avoid or reduce personnel gathering activities such as meetings and training as much as possible to reduce unnecessary going out.”
Voluntary Scheme Instrument	Psychological aid	Notice of the Ministry of Civil Affairs and the National Health Commission on in-depth Study and Implementation of the Spirit of General Secretary Xi Jinping’s Important Instructions to Further Improve the Emergency management of the Epidemic in Urban and Rural Communities	“Focus on personnel counseling and psychological comfort of closed management community”
Policy Target	Sub-policy Target	Text source	Extract Contents
Social Stability	Social order maintenance	Guiding Opinions of the General Administration of Market Regulation on Investigating and Punishing Illegal Acts of Raising Prices during the Emergency management of the Novel Coronavirus Infection	“To ensure the stability of the market price order of basic livelihood commodities during the emergency management of the novel coronavirus pneumonia epidemic, strengthen and standardize the market supervision departments at all levels to investigate and punish illegal acts of driving up prices”
Economic Recovery	Enterprise development and work resumption	Notice of the State Council on the Joint Emergency management Mechanism for Novel Coronavirus Emergency management Measures for Enterprises and Institutions Resuming Work and Production	“To guide the implementation of the various work requirements for the emergency management of the new crown pneumonia epidemic, and to promote the steady and orderly resumption of work and production of enterprises and institutions, this guide is specially formulated.”
Epidemic prevention and control	Welfare, team and fund support	Notice of the Ministry of Finance on Further Doing a Good Job in Guaranteeing Funds for the Emergency management of the Novel Coronavirus Pneumonia Outbreak	“By the unified deployment of the Party Central Committee and the State Council, timely response and decisive response should be made to guarantee the funding for orderly and effective epidemic emergency management.”

### 3.4 Social Network Analysis (SNA)

Social network is formed in terms of nodes and links [35]. The node represents the “social actors”, the link represents the “relationships between actors” [36]. China’s policy mix in response to COVID-19 can be regarded as a network structure, where nodes are the policy subjects, policy targets and policy instruments; the links are the relationships among the policy subjects, policy targets and policy instruments. Identifying key nodes and measuring network links are the focus of social network analysis (SNA) [34]. SNA is a set of mathematical methods and tools that visualize and examine the social dynamics among the network nodes by using various indicators such as density, centrality, concentration, etc. Several studies have demonstrated the viability of using SNA to understand the governance network structure and network dynamics in crises [37]. Therefore, SNA was adopted in this paper to reveal the dynamic of China’s response to COVID-19 policy mix. The measurement indicators used in this study mainly include density, network centralization, component, fragmentation and degree centrality. Specifically, density, network centralization, component, fragmentation were used to measure the network of “policy subject-policy subject”; degree centrality was used to measure the networks of “policy subject-policy subject”, “policy instrument-policy target” and “policy subject-policy instrument”. Degree centrality is a measure of the number of direct ties that nodes possess in the network [38], reflecting the importance and influence of nodes in the network. The greater the degree centrality of a network node, the closer the node is to the center of the network, and the more influential it is in the network [39].

## 4.0 Results

China’s policy mix in response to COVID-19 is a dynamic setting that shapes the evolution of the policy subjects, policy targets and policy instruments over time. In this study, the time dimension is introduced to identify the core policy subject, policy target, policy instrument and their influences in different periods, and then capture the dynamic evolution of China’s response to COVID-19 policy mix. Specifically, the entire duration of China’s response to COVID-19 was chopped into three time slices: T1 (January, 20, 2020-February 10, 2020); T2 (February 11, 2020-February 19, 2020); T3: (February 20, 2020-March 26, 2020). The periodic division is consistent with the important time points of COVID-19 emergency response in China. On January 20, 2020, the National Health Commission issued “Announcement No. 1” to include novel coronavirus pneumonia in the Class B infectious diseases stipulated by the “Infectious Disease Emergency management Law”, and adopt Class A emergency management measures. This marks the beginning of formal COVID-19 emergency response in China. In early February 2020, the COVID-19 broke out in full. To control the spread of virus, the central government of China intensified the formulation of COVID-19 emergency response policies. On February 19, the effect of epidemic prevention and control was obvious, the number of new cases showed a downward trend across China. In early March, many provinces in China reduced their PHE response levels. As of March 26, 2020, there were no new local cases in many provinces of China. China entered the normalization stage of COVID-19 emergency response, and the social economy began to restore in an orderly manner.

### 4.1 Core policy subjects and their evolutions in different phases

**Fig 4:**
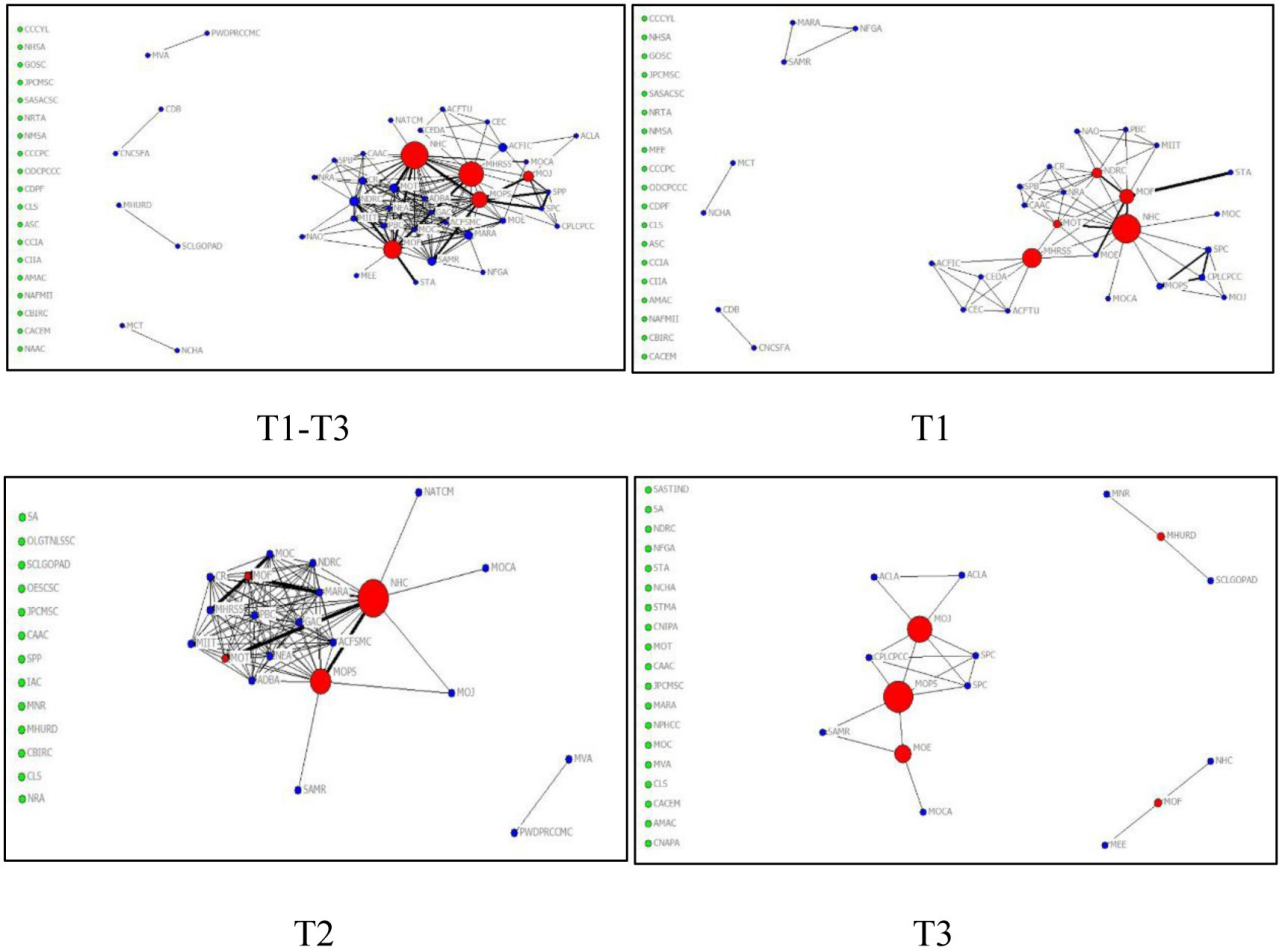
Policy subjects-policy subjects network topology diagram.

In this study, Netdraw program (version 6.212) was used to visualize the network topology structure of policy subjects in each time slice, as shown in Fig 4. In Fig 4, the red node represents the policy subjects with the top five degree centrality; the green node represents the policy subjects who issue policies independently; the blue node represents other policy subjects; the node size represents the size of degree centrality; the width of the line represents the strength of joint issuance among policy subjects.

In Fig 4, the core policy subjects and the relationships between subjects change with time in different stages of epidemic development. From T1 to T3 period, the total number of nodes in China’s response to COVID-19 policy subjects network show a downward trend, with more isolated nodes and obvious marginalization of nodes. In T1 phase, the number of nodes reaches the peak, and there is a strong joint policy issuance relationship among NHC, MOT and MOF. In T2 phase, the number of nodes decreases, but the closeness between policy subjects is significantly increased. This means that there are more joint policy issuing among policy subjects compared to T1 period. In T3 phase, the number of nodes is not much different, however, the interactions among nodes is significantly loose.

The change of the policy subjects network structure over time can also be described by statistics of the nodes, links, density, network centralization, component, and fragmentation, as shown in Table 3. The results imply that the “policy subjects network” of China’s response to COVID-19 presents a dynamic evolution characteristic. Overall, the policy mix consists of 86 policy subjects and 359 interactions. The nodes and links of the policy subjects network involved in each time slices are different. In T1 phase, the number of policy subjects (68) and links (127) between subjects is the largest. The number of policy subjects and the links between subjects shows a decreasing trend. In T3 phase, the number of nodes is the least (38), indicating that the number of policy subjects continues to decrease with the gradual improvement of relevant policies. According to the results of density, the density of policy subjects network maintains a relatively high level (39.86%) in the T2 stage. Stage T2 is a critical period of COVID-19 emergency response in China. During this period, multi-subject joint dispatch with different emergency resources and emergency capabilities is required to cope with epidemic. As time goes by, the network density declined and reached the lowest in T3 (2.84%) period. At this stage, the structure of policy subjects network is not centralized but the most sparse, indicating that the number of joint issuance among subjects decreases with the reduction of the urgency of epidemic emergency response. In terms of fragmentation, a similar evolution pattern as density was observed. The fragmentations of policy subject network in T1 (87.7%) and T3 (92.7%) periods were higher than that in T2 (69.3%) period, which manifested by isolated nodes and sparse connections between subjects. The result of network component showed that the number of sub-networks of policy subject network is large, and there were unconnected sub-networks and a large number of isolated nodes in each period. This indicates that the policy subject networks in T1, T2 and T3 periods were incomplete and vulnerable to future uncertainty. From the network centralization, the network of policy subjects is the most connected with each other in T2 period (18.09%), but relatively scattered in T1 (7.07%) and T3 (13.96%) periods. The joint issuance of policies among subjects was limited.

**Table 3:** Measurement of policy subjects network in four periods.

	All Terms (T1-T3)	Term1 (T1)	Term2 (T2)	Term3 (T3)
Policy Subjects (Nodes)	86	68	34	38
Interactions (Links)	359	127	197	40
Density	0.2902	0.1957	0.3986	0.0284
Network Centralization (%)	0.0924	0.0707	0.1809	0.1396
Component	48	41	15	25
Fragmentation (prop. of nodes that cannot reach each other)	0.836	0.877	0.693	0.927

To identify the core policy subjects of China’s response to COVID-19 at different periods, this study measured the degree centrality of the policy subjects. The results were listed in Table 4. The results show that the position and the influence of most policy subjects change over time. Only NHC (7.960, 27.273), MOF (6.965, 25.758), MOT (4.478, 22.727), and MHRSS (3.980, 22.727) have been at the center of the network during the T1 and T2 phases of the epidemic response. This indicates that the Chinese government attaches great importance to the role of financial, material and human resources in COVID-19’s response. Among them, NHC is an important functional department for the PHEs risk response. In the early stage of the epidemic outbreak, NHC led the establishment of the “Joint Emergency management Mechanism of the State Council” to organize and coordinate the entire response to COVID-19. NHC also actively issued medical and health technical specifications and emergency management plans to provide policy guidelines for the fight against the epidemic [40]. MOF invested special funds to guarantee the anti-epidemic materials and the resumption of work and production. MOT imposed traffic control to restrict population movement and opened green channels to ensure the transportation of critical medical and living supplies without disruption or delay. MHRSS stabilized the epidemic prevention personnel through employment subsidies and employment expansion.

**Table 4:** Top 5 policy subjects with highest NrmDegree centrality (%).

Policy Subjects	All Terms (T1-T3)	Term1 (T1)	Term2 (T2)	Term3 (T3)
NHC	10.588 (1)	7.960 (1)	27.273 (1)	
MOF	10.000 (2)	6.965 (2)	25.758 (2)	
MOPS	9.118 (3)		25.758 (2)	
MOT	7.647 (4)	4.478 (4)	22.727 (4)	
NDRC	7.647 (4)	5.473 (3)		
MOJ				16.216 (1)
MHRSS		3.980 (5)	22.727 (4)	
CPLCPCC				10.811 (3)
MOC			22.727 (4)	
MARA			22.727 (4)	
MOPS				16.216 (2)
SPC				10.811 (3)
SPP				10.811 (3)

Note: Numbers in parentheses represent the rank of the policy subject.

However, the core policy subjects at a certain stage do not occupy a dominant position in each time slice. With the development of COVID-19, the core policy subjects changed. In the T3 stage, the first level response to PHEs (the highest level of PHEs response in China) was lifted in most provinces of China, NHC, MOF, MOT and MHRSS was no longer located in the center of the network, MOJ (16.216) and MOPS (16.216) became the new core policy subjects. In the context of the initial victory of epidemic emergency response, to prevent illegal and criminal acts such as violations of public order and endangering public security, MOJ and MOPS strengthened the punishment of crimes to maintain social stability and promote economic recovery.

### 4.2 Core policy targets and their changes of in different phases

**Fig 5:**
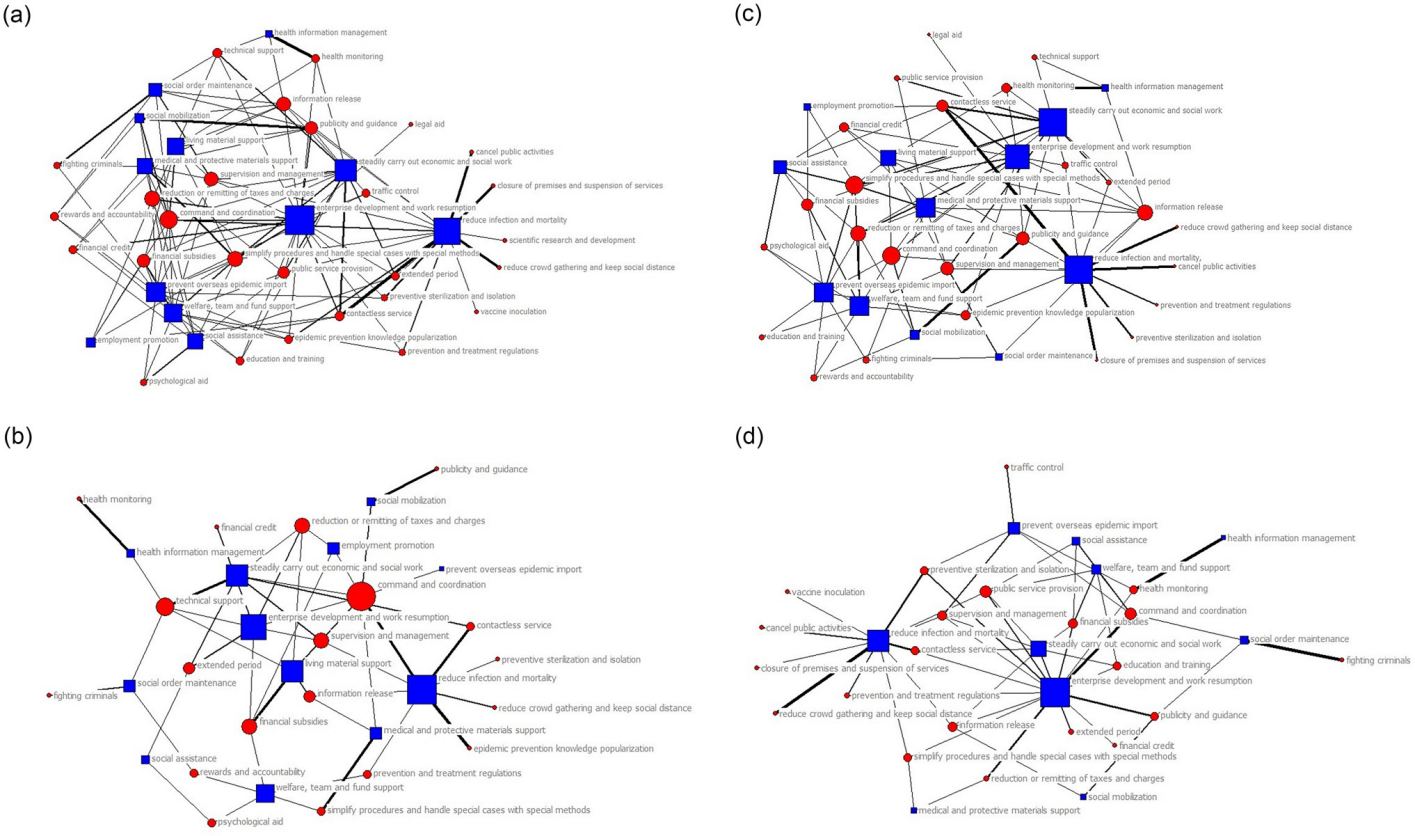
“Policy targets-policy instruments” network topology diagram.

In different periods, policy releases often have different focuses. By analyzing the core policy targets and their dynamic evolutions, it can reveal the priorities of China’s response to COVID-19 at different stages. Thus, 2-mode network topology diagram of “policy target-policy instrument” developed with NetDraw was presented in Fig 5. The red round node represents the policy instruments, the blue rectangular node represents policy targets; the node size represents the size of degree centrality; the width of the line represents the strength of the relationships between policy instruments and policy targets.

Based on Fig 5, it can be obtained that the core policy targets of China’s response to COVID-19 at different stages were different and evolved over time. And there are differences in the strongly linked policy instruments involved in the core policy targets. In the overall stage, instruments such as “contactless service”, “closure of premises and suspension of services”, “cancel public activities”, “reduce crowd gathering and keep social distance” and “preventive sterilization and isolation” jointly promoted the core policy target of “reduce infection and mortality”. Meanwhile, the policy instrument of “contactless service” serves the three core policy targets of “enterprise development and work resumption”, “steadily carry out economic and social work” and “reduce infection and mortality” at the same time. These characteristics can also be observed in T1 stage. In addition, in T2 stage, the policy instruments strongly associated with the core policy target of “reduce infection and mortality” include “contactless service”, “command and coordination” and “epidemic prevention knowledge popularization”. Entering the T3 stage, “enterprise development and work resumption” became the new core policy target, and “reduction or remitting of taxes and charges”, “command and coordination” and “publicity and guidance” were strongly related policy instruments for achieving this target. These results illustrated that the core policy target is different in different stages, and the realization of the core policy target depends on various policy instruments.

To explore the core policy targets and their dynamic evolutions of China’s response to COVID-19, the degree centrality of the policy targets in different time slices was performed, as shown in Table 5. It can be obtained from Table 5 that the policy target shows a certain continuity across time in T1-T3 stages of China’s response to COVID-19, and there are differences in core policy target. In stage T1, China’s main policy concern is “reduce infection and mortality” (0.520) and “steadily carry out economic and social work” (0.520). This is a period characterized by significant public health challenges and severe economic stress. The Chinese government emphasized that under the premise of scientific epidemic prevention, it is necessary to maintain the stable economic growth of development and make reasonable policy adjustments based on the epidemic development. “Medical and protective materials support” (0.360) also occupies an important position in T1 stage. During T1 period of the epidemic concentrated outbreak, the number of confirmed cases of COVID-19 in China increased sharply, while medical professionals and supplies were scarce. Especially in Wuhan, the “epicenter” of the virus in China, there was a medical surge. To alleviate this dilemma, the Chinese government quickly built specialty field hospitals (e.g. “Huoshenshan”, “Leishenshan” hospitals) and mobile cabin hospitals [41]. And “paired assistance” was released to mobilize the other 19 provinces to provide medical personnel and supplies to support Wuhan, which expanded Wuhan’s medical treatment capacity [5]. This implies that the key to response to PHEs lies in the proper distribution of emergency medical supplies, which is vital for reducing deaths and increasing the success rate of treatment. However, when COVID-19 broke out, the majority of countries were unprepared. The resources used to deal with the virus, especially personal protective equipment (PPE) (e.g. masks, medical protective clothing, etc.) and medical equipment (e.g. ventilators, ECMO, etc.) are seriously inadequate [2]. To effectively respond to the similar “Black Swan events” in the future, the government needs to heighten its surge capacity by assessing the scope of the crisis, mobilizing personnel and matching resources with the scale of the emergencies [42].

**Table 5:** Degree centrality (%) measures of policy targets.

Policy Targets		All Terms (T1-T3)	Term1 (T1)	Term2 (T2)	Term3 (T3)
Economic Recovery	Enterprise development and work resumption	0.704 (1)	0.440 (3)	0.368 (2)	0.714 (1)
	Employment promotion	0.185 (10)	0.120 (10)	0.158 (6)	-
	Steadily carry out economic and social work	0.519 (3)	0.520 (1)	0.316 (3)	0.333 (3)
Epidemic Prevention and Control	Reduce infection and mortality	0.630 (2)	0.520 (1)	0.421 (1)	0.524 (2)
	Prevent overseas epidemic import	0.444 (4)	0.360 (4)	0.053 (12)	0.238 (4)
	Welfare, team and fund support	0.407 (5)	0.360 (4)	0.263 (5)	0.190 (5)
	Social mobilization	0.185(10)	0.160 (9)	0.105 (9)	0.095 (8)
	Health information management	0.111 (12)	0.120 (10)	0.105 (9)	0.048 (10)
	Medical and protective materials support	0.333 (7)	0.360 (4)	0.158 (6)	0.095 (8)
Social Stability	Living material support	0.370 (6)	0.280 (7)	0.316 (3)	-
	Social assistance	0.333 (7)	0.240 (8)	0.105 (9)	0.143 (6)
	Social order maintenance	0.259 (9)	0.120 (10)	0.158 (6)	0.143 (6)

In T2 phase, the position of “reduce infection and mortality” (0.421) as the central policy target did not significantly change. At this stage, the epidemic has not been fully controlled, the first level response mechanism for PHEs has not been lifted, thus the “reduce infection and mortality” was still the core policy target. However, the core policy target was partially transferred to “enterprise development and work resumption” related to economic recovery. This is because after the end of the Chinese Lunar New Year holiday, some key enterprises with great impacts on people’s livelihood were allowed to resume production to ensure the supply of anti-epidemic materials and living materials.

In T3 phase, the core policy target of China’s response to COVID-19 changed, which shifted to “enterprise development and work resumption” (0.714). At this stage, the epidemic has been controlled, and attention of the government policy has turned to the resumption of work and production. Consequently, a large number of relevant safeguard policies were promulgated. Moreover, “prevent overseas epidemic import” (0.238) has also become one of the important policy targets at T3 stage. With the past of the epidemic peak in China and the large-scale spread of the epidemic in other countries, the focus of the policy target has shifted from domestic to abroad. This indicates that the changes in the core policy target are highly correlated with urgent issues in a specific period.

### 4.3 Core policy instruments and their variations of in different phases

**Fig 6:**
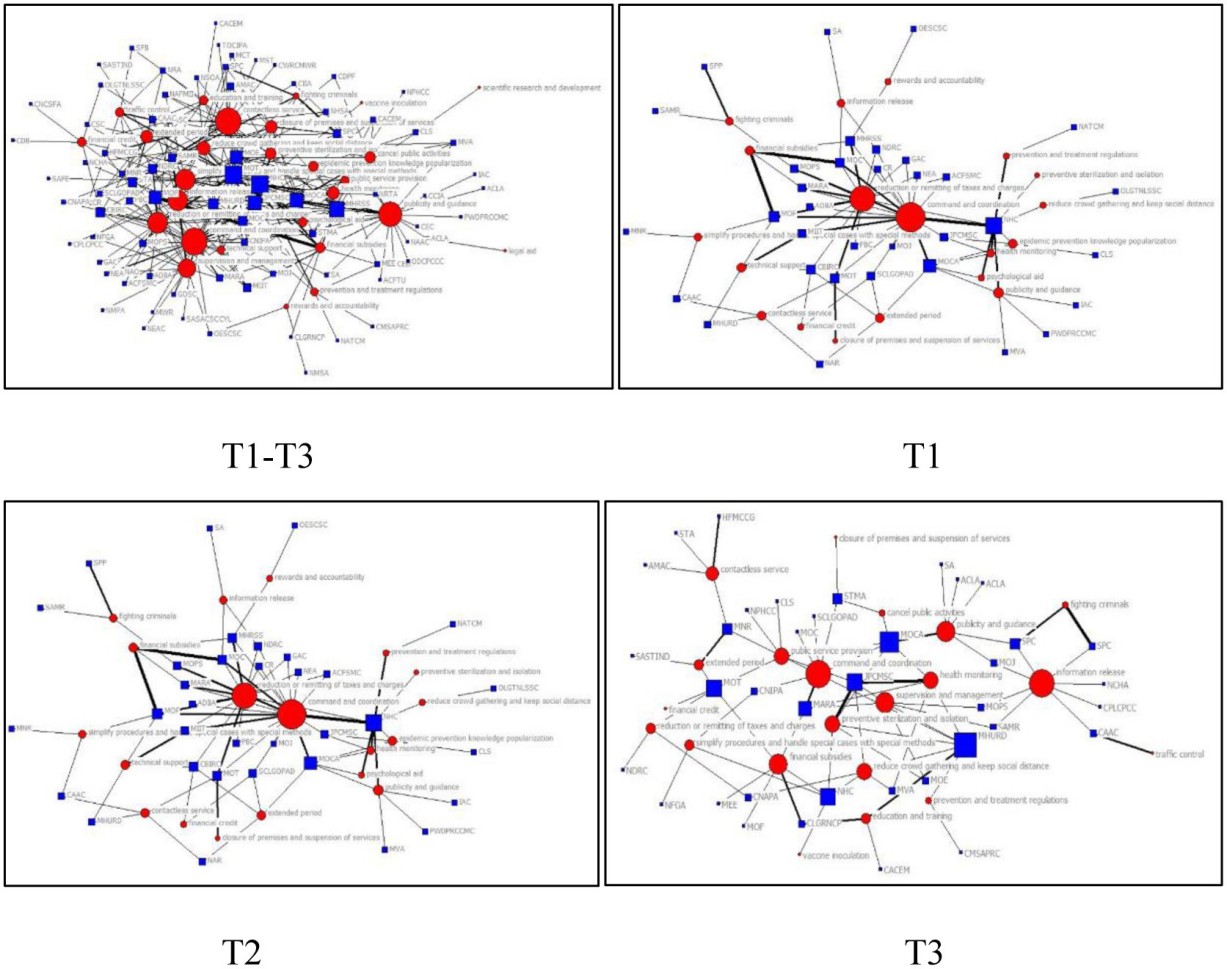
Policy subjects-policy instruments 2-mode network topology diagram.

To visually present the core policy instruments and their dynamic evolution of China’s response to COVID-19 in different periods, this study used NetDraw program to generate the 2-mode network topology of the “policy instrument-policy subject”, as displayed in Fig 6. The red round node represents the policy subjects, the blue rectangular node represents the policy instruments; the node size represents the size of degree centrality; the width of the line represents the strength of the relationship between the policy instruments and policy subjects.

From Fig 6, the whole process and each time period of COVID-19 emergency response in China involve the complex relationships between policy subjects and policy instruments. Multiple policy subjects adopted the same policy instrument and the policy subject adopting the same policy instrument in different stages was different, showing an obvious dynamic characteristic. For example, the policy instrument of “financial subsidies” was frequently used by policy subjects of MOF, MOE, NHC, MHRSS in T1 period. As the crisis changes, the policy subject adjusted the choice of policy instrument accordingly. In T2 period, “financial subsidies” was mainly used by MOF and MOC. During T3 period, MOT joined and became a new policy subject employing the policy instrument of “financial subsidies”. This reflects the dynamics and complexity of the use of policy instruments in response to the epidemic. Furthermore, based on Fig 6, it can also be intuitively seen that the connection between the policy instruments of “mandatory administration”, “economic incentive instruments” and the policy subject is thick, while the connection between “voluntary plan instruments”, “health promotion instruments” and the policy subject is relatively thin.

To study the evolution of policy instruments in China’s response to COVID-19 and to identify the core policy instruments in each time slice, this paper calculated the degree centrality of the policy instruments based on the “policy instrument-policy subject” network, as shown in Table 6. According to Table 6, the core policy instrument of COVID-19 emergency response in China changes over time, depending on the development of the virus. Overall, in T1-T3 phases, the sub-policy instrument of “publicity and guidance” (0.262, 0.147, 0.158) in “voluntary plan instruments” has a high degree centrality and is at the core position in the network. This reflects that China attaches great importance to the propaganda of epidemic prevention. Various government departments improve people’s public health literacy (e.g. washing hands, wearing masks, keeping social distancing) and encourage citizens to actively participate in the fight against the virus through education, training, policy interpretation, etc [15].

**Table 6:** Top 5 policy instruments with highest degree centrality (%).

Policy Instruments		All Terms (T1-T3)	Term1 (T1)	Term2 (T2)	Term3 (T3)
Mandatory Administration Instruments	Command and coordination	0.321 (1)	-	0.559 (1)	0.211 (1)
	Information release	0.250 (4)	0.169 (4)	-	0.211 (1)
	Supervision and management	-	0.154 (5)	-	0.158 (3)
	Closure of premises and suspension of services	-	0.154 (5)	-	-
Economic Incentive Instruments	Contactless service	0.321 (1)	0.323 (1)	0.118 (4)	-
	Reduction or remitting of taxes and charges	0.238 (5)	-	0.471 (2)	-
	Simplify procedures and handle special cases with special methods	0.238 (5)	0.246 (3)	-	-
	Financial subsidies	-	-	0.118 (4)	0.158 (3)
	Extended period	-	-	0.118 (4)	-
	Technical support	-	-	0.118 (4)	-
Voluntary Plan Instruments	Publicity and guidance	0.286 (3)	0.262 (2)	0.147 (3)	0.158 (3)
Health Promotion Instruments	Epidemic prevention knowledge popularization	-	-	0.118 (4)	-

Note: Numbers in parentheses represent the rank of the policy instrument.

In T1 phase, the most central policy instrument is “contactless service” (0.323). “Contactless service” handles business through a variety of off-site and non-contact methods such as online, self-service platform, mail, etc. This policy instrument cuts off the channel of virus transmission by creating spatial distance. As proposed by Liu et al (2020b), reducing social interaction and increasing physical distance are core parts of many public health policy instruments [43]. Moreover, COVID-19 pandemic is not only a public health crisis, but also brought catastrophic economic damage. The choice of public health policy instruments should be a multi-objective optimization issue that requires a trade-off between the largest social distance and the smallest economic loss [44, 45]. “Contactless service” is such a policy instrument, which has an advantage in limiting the spread of virus and maintaining the basic operation of the society.

In T2 phase, the role of “technical support” (0.118) policy instrument is prominent. An emerging trend during the COVID-19 pandemic is that governments of various countries use advanced technologies, such as artificial intelligence (AI), cloud computing, big data, to conduct extensive contact tracking to identify and isolate risk groups [46]. So does the Chinese government. In China, the typical product supported by “technical support” policy instrument is the “Health Code”. “Health Code” is a useful assistant to track the outdoor route of confirmed cases and quickly identify close contacts [15]. Although this may lead to a debate about whether personal privacy and human rights are violated, these measures do help to effectively contain the spread of the virus promptly [41]. The effectiveness of “technical support” has also been verified by COVID-19 response experience in South Korea. South Korea used big data and AI technology to build the “COVID-19 smart management system”, which improved the response efficiency of the pandemic [24].

In T3 phase, “information release” (0.211) became one of the new core policy instruments for China’s response to COVID-19. The “information release” policy instrument is mainly used to strengthen the risk communication with various groups in the form of real-time information, treatment plans and work resumption guidance on COVID-19. Chinese government highlights the importance of timely and authentic information communication and disclosure in fighting against the COVID-19, which is most expected by the public. As indicated by Tyler (1988), if citizens believe that the threat is real and understand the policy logic behind the decision, they seem to be more willing to accept policy measures, even the harshest ones [47]. However, lack of information transparency and the dissemination of false information may weaken the public trust in public institutions and cause lasting damage. The failure of the United States early response to COVID-19 was partly due to the insufficient disclosure of epidemic-related information and the crisis of public trust in the Trump administration [48].

In addition, the core policy instrument of China’s response to COVID-19 changed to “command and coordination” in T2 and T3 phases, which belongs to mandatory administration instruments. In light of the high uncertainty, unpredictability and complexity of COVID-19 crisis management, the Chinese government adopted the policy instrument of “command and coordination” to strengthen the overall planning of various resources such as human, financial and material and unify regulations on social production activities and epidemic response behaviors. The “command and coordination” with government-centric forms a powerful “joint force” that concentrates all resources to defeat the epidemic in a short time. Practical experience in China has forcefully demonstrated the effectiveness of the “command and coordination” policy instrument. Meanwhile, the Chinese government’s adoption of this policy instrument is an authority-based consensus under the centralized regime [16]. Prior studies believed that compared to federal or decentralized governance structures, mandatory administration policy instrument seems to work more effectively and face less institutional friction during the pandemic crisis in centralized regimes [45]. Moreover, in T2 and T3 phases, the “financial subsidies” policy instrument also has high degree centralities (0.118, 0.158) and is at the core of the network. The “financial subsidies” policy instrument is simple and direct, which ensures the quick and efficient supply of funding and medical supplies for China’s response to COVID-19. In T2 period, sufficient financial funds are required for testing, quarantine, isolation and treatment. In T3 period, the spread of the virus was controlled. The policy instrument of “financial subsidies” turned to providing support for economic recovery, especially for the worst-hit industries, such as aviation and transportation [44].

## 5.0 Discussion and policy implications

### 5.1 Evolutions of core policy subjects in response to COVID-19

The results of this research implied that there are few joint policy issuances among subjects of COVID-19 emergency response in China, to develop policy cooperation space is urgent. At present, policy subjects are more independent to issue policies through institutionalized functional division of government departments, rather than the full collaboration and partnership among departments. However, the complexity of the PHEs governance determines that there is a functional intersection between policy subjects with different emergency resources and capabilities. And a society’s crisis response capability largely depends on the breadth and depth of the relationships between responders [49]. Therefore, interorganizational collaboration (IOC) becomes an important mechanism to address challenging public health problems. Government should strengthen the joint issuance of policy subjects and establish a cross-sectoral to policy formulation and release platform to deal with the threat of pandemic.

In addition, the research results highlighted that the China’s policy mix in response to COVID-19 involves multiple policy subjects and evolves over time. The diversification of policy subjects not only provides resources and information convenience for epidemic response, but also increases the difficulty of collaboration among subjects posed different cultural and administrative contexts [50]. Therefore, it is recommended that the government should clarify the core policy subjects during different periods of the pandemic. As found in this study, the finance department should be emphasized as the policy subject to ensure the funding and materials for public health crisis response, apart from the traditional Ministry of Health. This view is confirmed by the research of Coccia (2020), which highlights that an effective strategy to address the threat of a pandemic is fiscal sector to increase health spending and R&D investment to support a sustained policy response [51].

### 5.2 Changes of core policy targets in response to COVID-19

The research results showed that clear phased policy target is the typical characteristic of China’s policy mix to deal with COVID-19. Some studies claimed that the recurrence of the COVID-19 in some countries lied in unclear policy targets [48]. Before the epidemic crisis was completely controlled, the premature activation of economic policy targets such as the resumption of work and production disrupted the epidemic response steps and prolonged the epidemic emergency response cycle. In a highly complex and uncertain crisis such as COVID-19, the policy targets are multiplied and cross-appear at different stages. Therefore, the government should arrange the priorities of policy objectives in a timely and reasonable manner.

Moreover, the core policy target in China’s COVID-19 policy mix is dynamically developing. This reflects the contingency perspective of policy theory, which believes that the formulation of policy targets should adjusted with the environment changes [52]. Therefore, faced with a pandemic, the important thing is to find the core policy goals “matching” with the specific circumstances to improve the efficiency of crisis response.

### 5.3 Variation of policy instruments in response to COVID-19

The results of the study showed that China attaches importance to the combined application of policy instruments in fight against the COVID-19. Facts have shown that it is the combined use of these policy instruments that have enabled China to achieve excellent achievements in the fight against the COVID-19. Thus, the combination use of multiple policy instruments is critical to respond to such a highly infectious virus. As some previous studies suggested that the effectiveness of any single policy instrument is limited and cannot offer effective solutions to PHEs [53]. Policy instruments can produce positive effects only when fit together [54]. For example, in the absence of effective treatment schemes and vaccines, the Chinese government preferred to impose some straightforward yet possibly unpopular “mandatory administration instruments” and “economic incentive instruments” to deal with the challenge of COVID-19. The experience of other countries, especially South Korea and Singapore, has also confirmed the effectiveness of these policy instruments [22, 29]. Therefore, “mandatory administration instruments” and “economic incentive instruments” could be implemented together to play a direct and efficient role in fight against pandemic.

However, some studies argued that the successful response to COVID-19 in East Asian countries is largely due to their “tight culture”, which presents a strong willingness to obey and cooperate [55]. Therefore, given the cultural, political and historical differences in various countries, the effectiveness of the public health intervention policy instruments may also change in the broader environment [12]. The policymakers should be cautious when implementing these policies. Meanwhile, there is a view that “mandatory administration instruments” and “economic incentive instruments” are too rigid and may lack flexibility if fully dependent [56]. Therefore, it is recommended that policy makers should use “health promotion instrument” and “voluntary plan instrument” in balance to reduce the negative impact of pandemics on social and economic costs.

## 6.0 Conclusions

Policy is the most direct means for the government to resolve the crisis. This paper studied the core node and dynamic evolution of China’s policy mix in response to COVID-19 from the perspective of network. An analysis framework of “policy subject-policy target-policy instrument” was constructed firstly. Then, based on the policy text data, social network analysis (SNA) was used to analyze the core node and dynamic of China’s policy mix in response to COVID-19 in different stages of the crisis. The results showed that the policy subject, policy target, and policy instrument are distinctive in different stages and change with time. (1) The core policy subjects mainly involve NHC, MOF, MOT, and MHRSS. Policy subjects issue policies more independently rather than jointly. (2) The overall policy target is mainly “epidemic prevention and control” and “economic recovery”. However, there are differences in different periods. In early stage, “reduce infection and mortality” and “steadily carry out economic and social work” were the core policy targets; in middle stage, “reduce infection and mortality” was the core policy target, supplemented by “enterprise development and work resumption”; and then the core policy target transformed to “enterprise development and work resumption” in last stage. (3) Policy instrument in China’s response to COVID-19 is characterized by dynamic evolution and unbalanced distribution. Core policy instruments include “contactless service”, “command and coordination”, “information release”, “financial subsidies” and “technical support”. Policy instruments of “mandatory administration instruments” and “economic incentive instruments” were used frequently, while “health promotion instruments” and “voluntary plan instruments” were used insufficiently. Based on these findings, this research suggested that the government should play the pivotal role of core policy subjects and strengthen the joint issuance with marginal subjects; clarify the core policy targets at different stages and adjust them dynamically; adopt differentiated policy instruments and optimize the combination of policy instruments.

This study presents critical contributions to theory, method and practice. In theory, an integrating three-dimensional analysis framework covering policy subject, policy target, and policy instrument was developed, which can serve as an analysis tool for similar policy research. This research also extends the researchers’ earlier efforts in PHEs policy analysis by moving beyond the focus on a single policy instrument to broader policy mixes and their interactions. In method, this study provides a replicable method for policy research by combining SNA technology with qualitative and quantitative data. This attempt enables a better understanding of the dynamic of China’s COVID-19 policy mix. In practice, this research contributes to the practitioners formulate effective public policies. This study provides the strategic information for policymakers to improve the accuracy of matching policy subject, policy target and policy instrument at different stages by studying the dynamic evolution of China’s COVID-19 policy mix based on time slices. And a series of core policy targets and policy instruments determined in this research also provide references for policymakers to identify the key tasks and deploy the core policies in different stages of response to PHEs. Moreover, China’s experiences and lessons in fighting against COVID-19 may produce administrative insights or policy implications for other settings. Other nations can learn from China’s successful practices and implement appropriate responses in pandemic situations in light of their national conditions.

There are still some limitations and thus calls for further research. First, data were collected through manual textual analysis. Manual text interpretation relying on researchers’ prior knowledge is becoming more and more unsustainable in terms of operation cost, especially in the era of big data. In the future, computer technology (e.g. data crawler, big data analytics) is recommended to be adopted in textual analysis to solve this problem. Secondly, the research sample is limited to policy documents at the central level. However, there are differences in the outbreak and development of COVID-19 in various provinces of China. This paper regarded China’s response to COVID-19, which may lead to the neglect of some local practice and policy differences. It is suggested that future studies could include local government documents for comparative research. Thirdly, this study is based on a single cultural context of China, the research results may not be fully applicable to all regions/countries affected by COVID-19. It is recommended to test the transferability of this study’s findings to other nations with different institutional structures and cultural orientations in future research. In addition, expanding the geographic focus beyond China and conducting cross-cultural comparative research is also an important direction in the future.

## Supporting information

S1 Appendix Materials-organizations issued the policies. (DOCX)

S1 Dataset (XLSX)
