# Correction: Dynamic evolution of policy mix in response to COVID-19: Practice from China

**DOI:** 10.1371/journal.pone.0296604

**Published:** 2024-01-02

**Authors:** Lei Du, Wei Lu

There are errors in the Funding section. The correct Funding statement is: This work was supported by [Youth Fund Project of Humanities and Social Sciences of the Ministry of Education, China (Grant numbers 21XJC630002)]; [2022 Project of the Research Center for Social Development and Social Risk Control of the Key Research Base of Philosophy and Social Sciences in Sichuan Province (Grant numbers SR22A04)]; [2023 Chengdu Philosophy and Social Science Planning Project (Grant numbers 2023BS125)]; [Fundamental Research Funds for the Central Universities of Southwest Minzu University (Grant numbers 2023SQN09)]. The funders had no role in study design, data collection and analysis, decision to publish, or preparation of the manuscript.
