# Supplementary material for: Dynamic evolution of policy mix in response to COVID-19: Practice from China
Source: PLoS One. 2023 Sep 28;18(9):e0291633. doi: 10.1371/journal.pone.0291633 (PMC10538659; doi:10.1371/journal.pone.0291633)
Supplement: S1 Appendix — (DOCX) [file pone.0291633.s001.docx]

**S1 Appendix, Materials-Organizations Issued the Policies**

| Organizations Name | Organization abbreviation |
| --- | --- |
| Ministry of Finance | MOF |
| Ministry of Education | MOE |
| Ministry of Industry and Information Technology | MIIT |
| Central Committee of the Communist Youth League | CCCYL |
| National Development and Reform Commission | NDRC |
| State Taxation Administration | STA |
| National Health Commission | NHC |
| Commission for Politics and Law of the Communist Party of China Central Committee | CPLCPCC |
| Supreme People's Court | SPC |
| Supreme People's Procuratorate | SPP |
| Ministry of Justice | MOJ |
| Ministry of Public Security | MOPS |
| National Healthcare Security Administration | NHSA |
| General Office of the State Council | GOSC |
| Joint Emergency management Mechanism of the State Council | JPCMSC |
| State-owned Assets Supervision and Administration Commission of the State Council | SASACSC |
| Ministry of Transport | MOT |
| Ministry of Civil Affairs | MOCA |
| All China Lawyers Association | ACLA |
| All China Federation of Industry and Commerce | ACFIC |
| Ministry of Human Resources and Social Security | MHRSS |
| All China Federation of Trade Unions | ACFTU |
| China Enterprise Directors Association | CEDA |
| China Enterprise Confederation | CEC |
| Agricultural Development Bank of China | ADBA |
| All China Federation of Supply and Marketing Cooperatives | ACFSMC |
| General Administration of Customs | GAC |
| Ministry of Commerce | MOC |
| National Audit Office | NAO |
| State Administration for Market Regulation | SAMR |
| National Radio and Television Administration | NRTA |
| National Mine Safety Administration | NMSA |
| Ministry of Ecology and Environment | MEE |
| Central Committee of the Communist Party of China | CCCPC |
| Organization Department of the CPC Central Committee | ODCPCCC |
| China Disabled Persons' Federation | CDPF |
| China Law Society | CLS |
| Accounting Society of China | ASC |
| China Construction Industry Association | CCIA |
| China Institute of Internal Audit | CIIA |
| Asset Management Association of China | AMAC |
| National Association of Financial Market Institutional Investors | NAFMII |
| China Banking and Insurance Regulatory Commission | CBIRC |
| China Association of Construction Enterprise Management | CACEM |
| People's Bank of China | PBC |
| National Archives Administration of China | NAAC |
| China Scholarship Council | CSC |
| National Ethnic Affairs Commission | NEAC |
| National Energy Administration | NEA |
| State Administration of Foreign Exchange | SAFE |
| State Tobacco Monopoly Administration | STMA |
| China National Intellectual Property Administration | CNIPA |
| Trademark Office of the China Intellectual Property Administration | TOCIPA |
| State Council Leading Group Office of Poverty Alleviation and Development | SCLGOPAD |
| National Railway Administration | NRA |
| Civil Aviation Administration of China | CAAC |
| State Post Bureau | SPB |
| China Railway | CR |
| Ministry of Science and Technology | MST |
| National Social Organization Administration | NSOA |
| China National Centre for Student Financial Aid | CNCSFA |
| China Development Bank | CDB |
| National Forestry and Grassland Administration | NFGA |
| Ministry of Agriculture and Rural Affairs | MARA |
| Ministry of Water Resources | MWR |
| Ministry of Veterans Affairs | MVA |
| Political Work Department of People’s Republic of China Central Military Commission | PWDPRCCMC |
| Ministry of Culture and Tourism | MCT |
| National Cultural Heritage Administration | NCHA |
| Changjiang Water Resources Commission of the Ministry of Water Resources | CWRCMWR |
| China Securities Regulatory Commission | CSRC |
| Housing Fund Management Center of the Central Government | HFMCCG |
| Ministry of Housing and Urban-Rural Development | MHURD |
| National Medical Products Administration | NMPA |
| Standardization Administration | SA |
| National Administration of Traditional Chinese Medicine | NATCM |
| Office of the Leading Group for the Third National Land Survey of the State Council | OLGTNLSSC |
| Office of the Education Steering Committee of the State Council | OESCSC |
| Insurance Association of China | IAC |
| State Administration of Science, Technology and Industry for National Defence | SASTIND |
| Chinese National Association of Patent Agents | CNAPA |
| China Maritime Safety Administration of the People’s Republic of China | CMSAPRC |
| Central Leading Group on Responding to the Novel Coronavirus Pneumonia | CLGRNCP |
| Ministry of Natural Resources | MNR |
| China Association of Construction Enterprise Management | CACEM |
| National Patriotic Health Campaign Committee | NPHCC |
